# Endoscopic submucosal dissection of a rectal lesion using a new flower-shaped multiband traction system

**DOI:** 10.1055/a-2563-1388

**Published:** 2025-04-03

**Authors:** Fausto Riu Pons, Marta Antón Arnal, Miguel Angel Pantaleón Sanchez, Marc Batlle Garcia, Agustín Seoane Urgorri, Xavier Bessa Caserras, Luis Barranco Priego

**Affiliations:** 1 16548 Gastroenterology Department and Endoscopy Unit, Hospital del Mar, Barcelona, Spain; 2 16551 Hospital del Mar Medical Research Institute, Barcelona, Spain

Endoscopic submucosal dissection (ESD) is the preferred treatment for en bloc resection of tumors of the superficial gastrointestinal tract. This complex technique carries a high risk of complications and is time consuming. Maintaining a firm submucosal space through tissue traction is key for a precise and safe dissection 1 . There is a growing interest in the development of new traction devices to facilitate ESD procedures 2 3 .

We designed a novel traction technique using five elastic bands. Four of these are attached to a central band with a thread knot, creating a flower-like appearance. A crossed thread over the central band provides a handle for the entire piece ( Fig. 1 ).

**Fig. 1:**
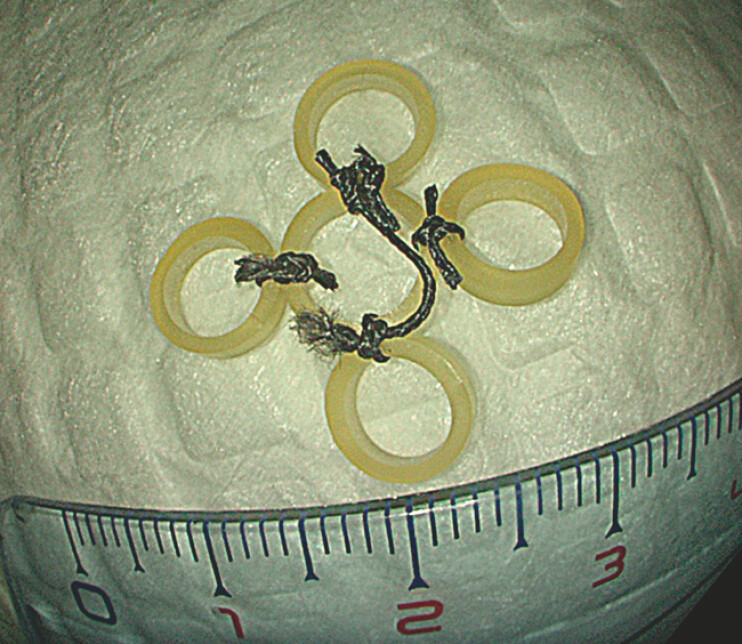
The flower-shaped multiband traction system.

A 64-year-old woman with a 50-mm laterally spreading tumor (LST)-granular nodular mixed type, located in the rectum (JNET type 2B) ( Fig. 2 ), was referred for endoscopic resection. After submucosal injection of succinylated gelatin mixed with indigo carmine, we performed ESD with a DualKnife-J (Olympus, Tokyo, Japan) ( Video 1 ). Once the initial circumferential incision and trimming was completed, each peripheral band was anchored with a clip to the four quadrants of the flap ( Fig. 3 ). This first step improved the dissection plane through centripetal traction. When tension was lost, the central band was anchored with a clip through the thread to the contralateral wall of the rectum. This second step enabled dynamic traction, enhancing the endoscopic view and allowing dissection to be completed ( Fig. 4 , Fig. 5 ). The procedure was performed without complications, and the patient was discharged the next day.

**Fig. 2:**
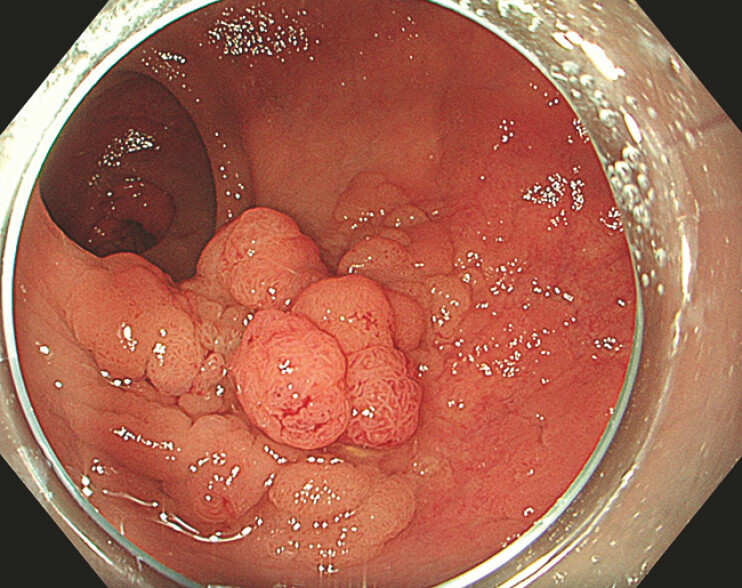
White-light imaging of a 50-mm rectal lesion (0-IIa+Is, laterally spreading tumor-granular nodular mixed type).

**Fig. 3:**
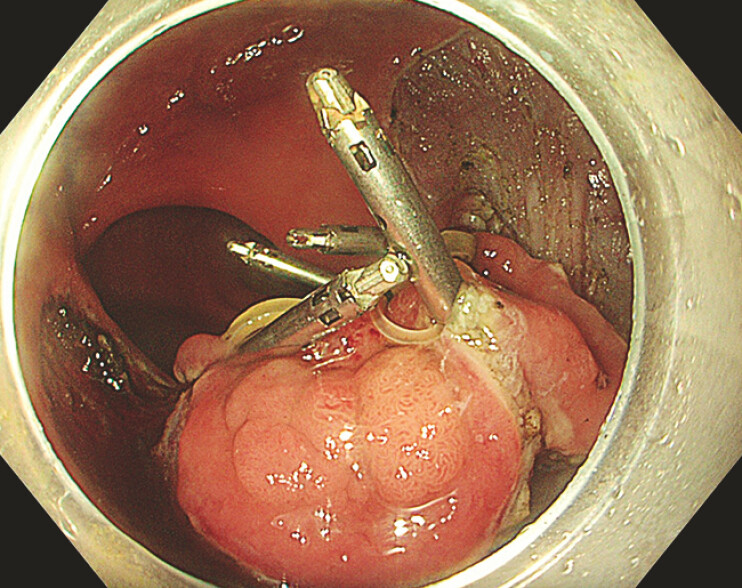
The multiband traction system creates an initial centripetal traction.

**Fig. 4:**
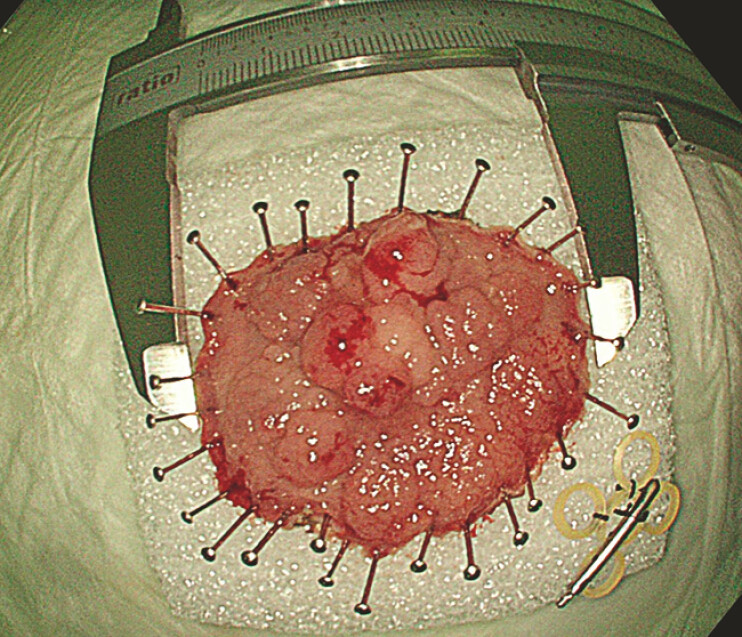
Macroscopic view of the resected lesion.

**Fig. 5:**
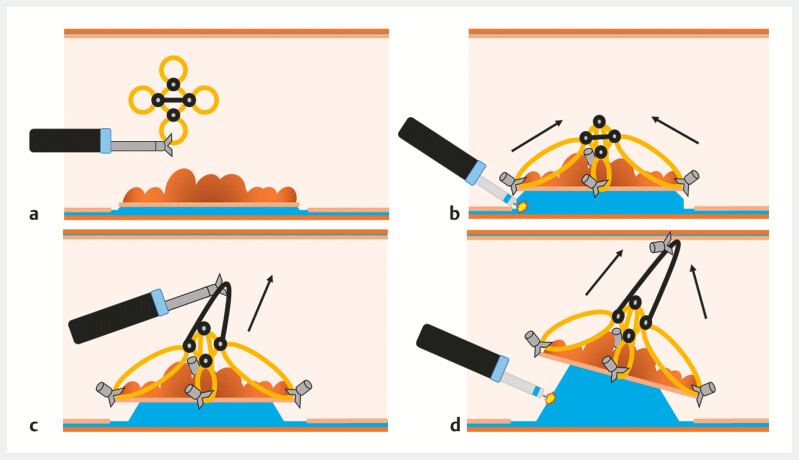
Illustration of the use of the traction device. **a** The flower-shaped multiband traction system is inserted after circumferential incision of the lesion. **b** The four bands are anchored with clips onto the flap, creating centripetal tension. **c** When tension is lost, the crossed thread over the central band is anchored by another clip attached to the contralateral wall of the rectum. **d** This provides global traction for the entire piece, enhancing the endoscopic view and facilitating the completion of the dissection.

Endoscopic submucosal dissection of a rectal lesion assisted by a flower-shaped multiband traction system.Video 1

Pathological examination revealed a pT1 adenocarcinoma with no risk factors. Following a multidisciplinary decision, this was considered a curative resection.

In conclusion, the flower-shaped multiband traction system is a simple traction technique that could optimize the safety and duration of ESD. This strategy seems feasible for LSTs with no bulky component and good maneuverability. Further studies are needed.

Endoscopy_UCTN_Code_TTT_1AQ_2AD_3AD

## Notes

Endoscopy E-Videos https://eref.thieme.de/e-videos
*E-Videos* is an open access online section of the journal *Endoscopy* , reporting on interesting cases and new techniques in gastroenterological endoscopy. All papers include a high-quality video and are published with a Creative Commons CC-BY license. Endoscopy E-Videos qualify for HINARI discounts and waivers and eligibility is automatically checked during the submission process. We grant 100% waivers to articles whose corresponding authors are based in Group A countries and 50% waivers to those who are based in Group B countries as classified by Research4Life (see: https://www.research4life.org/access/eligibility/ ). This section has its own submission website at https://mc.manuscriptcentral.com/e-videos .
